# A Male with Unilateral Microphthalmia Reveals a Role for *TMX3* in Eye Development

**DOI:** 10.1371/journal.pone.0010565

**Published:** 2010-05-11

**Authors:** Ryan Chao, Linda Nevin, Pooja Agarwal, Jan Riemer, Xiaoyang Bai, Allen Delaney, Matthew Akana, Nelson JimenezLopez, Tanya Bardakjian, Adele Schneider, Nicolas Chassaing, Daniel F. Schorderet, David FitzPatrick, Pui-yan Kwok, Lars Ellgaard, Douglas B. Gould, Yan Zhang, Jarema Malicki, Herwig Baier, Anne Slavotinek

**Affiliations:** 1 Division of Genetics, Department of Pediatrics, University of California San Francisco, San Francisco, California, United States of America; 2 Department of Physiology, University of California San Francisco, San Francisco, California, United States of America; 3 Cardiovascular Research Institute, University of California San Francisco, San Francisco, California, United States of America; 4 Department of Biology, University of Copenhagen, Copenhagen, Denmark; 5 Departments of Ophthalmology, Anatomy and the Institute for Human Genetics, University of California San Francisco, San Francisco, California, United States of America; 6 Genome Sciences Center, BC Cancer Research Center, Vancouver, British Columbia, Canada; 7 Department of Dermatology, Cardiovascular Research Institute and Institute for Human Genetics, University of California San Francisco, San Francisco, California, United States of America; 8 Clinical Genetics Division, Albert Einstein Medical Center, Philadelphia, Pennsylvania, United States of America; 9 Service de Génétique Médicale, Université de Toulouse, Toulouse, France; 10 Institut de Recherche en Ophtalmologie, University of Lausanne and Ecole Polytechnique Fédérale de Lausanne, Lausanne, Switzerland; 11 Medical Research Council Human Genetics Unit, Western General Hospital, Edinburgh, United Kingdom; 12 Department of Ophthalmology, Harvard Medical School, Boston, Massachusetts, United States of America; University of Arkansas for Medical Sciences, United States of America

## Abstract

Anophthalmia and microphthalmia are important birth defects, but their pathogenesis remains incompletely understood. We studied a patient with severe unilateral microphthalmia who had a 2.7 Mb deletion at chromosome 18q22.1 that was inherited from his mother. *In-situ* hybridization showed that one of the deleted genes, *TMX3*, was expressed in the retinal neuroepithelium and lens epithelium in the developing murine eye. We re-sequenced *TMX3* in 162 patients with anophthalmia or microphthalmia, and found two missense substitutions in unrelated patients: c.116G>A, predicting p.Arg39Gln, in a male with unilateral microphthalmia and retinal coloboma, and c.322G>A, predicting p.Asp108Asn, in a female with unilateral microphthalmia and severe micrognathia. We used two antisense morpholinos targeted against the zebrafish *TMX3* orthologue, *zgc:110025*, to examine the effects of reduced gene expression in eye development. We noted that the morphant larvae resulting from both morpholinos had significantly smaller eye sizes and reduced labeling with islet-1 antibody directed against retinal ganglion cells at 2 days post fertilization. Co-injection of human wild type *TMX3* mRNA rescued the small eye phenotype obtained with both morpholinos, whereas co-injection of human *TMX3*(p.Arg39Gln) mutant mRNA, analogous to the mutation in the patient with microphthalmia and coloboma, did not rescue the small eye phenotype. Our results show that haploinsufficiency for *TMX3* results in a small eye phenotype and represents a novel genetic cause of microphthalmia and coloboma. Future experiments to determine if other thioredoxins are important in eye morphogenesis and to clarify the mechanism of function of *TMX3* in eye development are warranted.

## Introduction

Birth defects affect an estimated 120,000 (1 in 33) babies born in the United States each year, and are the leading cause of death in the first year of life [1]. Anophthalmia is characterized by the absence of an eye or the presence of a rudimentary eye, and has a prevalence of up to 30 cases per 100,000 individuals [2]. Anophthalmia is closely related to microphthalmia (small eye). Coloboma (failure of the choroid or optic fissure to fuse, also known as an optic fissure closure defect) frequently occurs together with microphthalmia and may have a similar pathogenesis to microphthalmia in some cases.

Mutations in several transcription factors that are expressed during eye development have been shown to cause anophthalmia, microphthalmia and coloboma [2]–[5]. The eye phenotype is thought to arise from several basic pathological mechanisms - a failure of lens formation (for example, *SOX2* and *PAX6* haploinsufficiency [6], [7]), a failure of optic vesicle formation or regression of the optic vesicle (for example, *RAX* loss of function [8]) and impaired retinal development (for example, *CHX10, OTX2* and *MITF* haploinsufficiency [9]). However, a significant proportion of patients with anophthalmia and microphthalmia, estimated to be more than 60–70%, do not have an identified genetic etiology for their birth defect, and it is highly likely that new genes and pathways remain to be discovered [2].

Array comparative genomic hybridization (array CGH) is an effective methodology for screening whole genomes for submicroscopic chromosome aberrations [10]. Array hybridization has also been used to study patients with congenital heart disease [11], cleft palate [12] and diaphragmatic hernia [13]–[15]. Our previous studies on diaphragmatic hernia patients identified a novel 18q22.1 deletion using the Affymetrix GeneChip® Human Mapping 100K Set in a patient with unilateral microphthalmia and right-sided diaphragmatic hernia [16]. The following work describes our evaluation of the genes contained within this deletion: Cadherin 19, type 2 preprotein, (*CDH19*; Accession number NM_021153), Thioredoxin domain-containing 10 (*TMX3*, also known as *TXNDC10*, Accession number NM_019022), Dermatan sulfate epimerase-like (*DSEL*; Accession number NM_032160), and Coiled-coil domain-containing 102B (*CCDC102B*; Accession number NM_001093729), for a role in the pathogenesis of the microphthalmia found in the propositus.

## Results

The 13-month old propositus with severe unilateral microphthalmia and diaphragmatic hernia has previously been described [16], [17]. The propositus's parents were apparently without birth defects, but were not examined by us personally, and a maternal aunt was reported to have anophthalmia, although she was unable to be examined. Sequencing of the *STRA6* (Stimulated-by-retinoic acid-6; Accession number NM_022369) gene that is mutated in individuals with a phenotype comprising anophthalmia, diaphragmatic defects, cardiac malformations and pulmonary agenesis, was performed on the propositus, and was negative [17].

**Figure 1:**
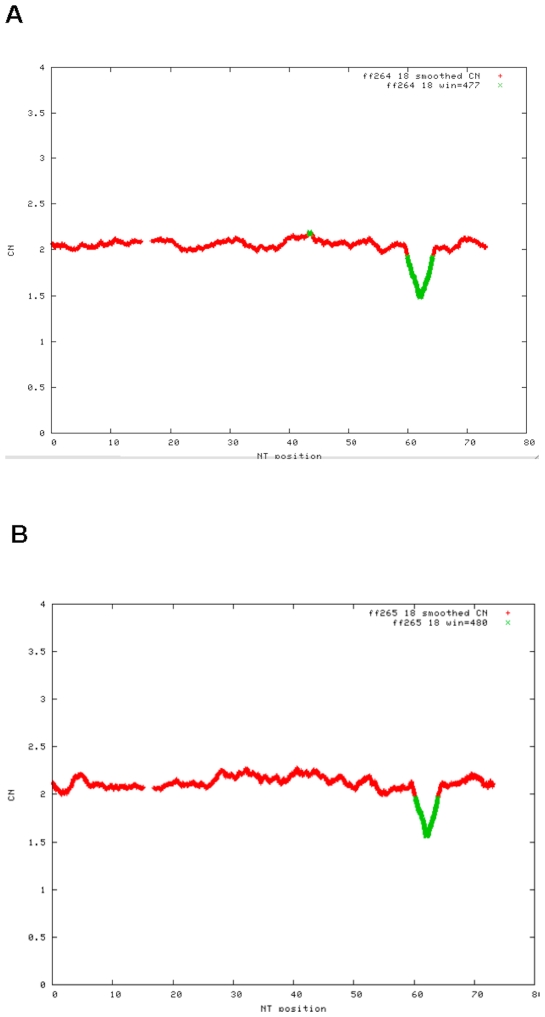
A 500K Microarray shows a 2.7 Mb deletion of 18q22.1 in the propositus. Fig. 1A. Graph of smoothed copy number for chromosome 18 from the Affymetrix 500K Array in the propositus, indicating loss of copy number (green color) and demonstrating a chromosome deletion at chromosome 18q22.1. The x-axis shows the nucleotide number from 1–76,117,153 on chromosome 18 and the y-axis shows smoothed copy number. Fig. 1B. Graph of smoothed copy number for chromosome 18 from the Affymetrix 500K Array in the mother of the propositus, indicating a similar loss of copy number (green color) at chromosome 18q22.1. The x-axis shows the nucleotide number from 1–76,117,153 on chromosome 18 and the y-axis shows smoothed copy number.

We used an Affymetrix GeneChip Human Mapping 500K Set to repeat our array studies in this family to refine the breakpoints of the 2.7 Mb 18q22.1 deletion that were previously identified using the Affymetrix GeneChip Human Mapping 100K Set [16]. The proband's deletion extended from SNP_A-4257584 at chr18:61,987,859 to SNP_A-1938047 at chr18:64,614,741 (base pairs numbered according to version hg18 of the UCSC Genome Browser, http://genome.ucsc.edu; Fig. 1A), inclusive, and his mother's deletion extended from SNP_A-4238202 at 62,058,576 to SNP_A-4257824 at 64,600,521, inclusive (Fig. 1B). The proband therefore has a slightly larger deletion than his mother based on these single nucleotide polymorphisms (SNPs), but both deletions contain the same genes. We did not find any other significant copy number variations (CNVs) in the propositus or in his parents (data not shown). The father did not have the same deletion using the same 500K array, and no other family members were available to be tested. The 18q22.1 deletion was confirmed by fluorescence in-situ hybridization (FISH) using bacterial artificial chromosome (BAC) probes RP11-246I7 and RP11-105L16, which were deleted [16].

The deleted region on chromosome 18 contains three genes that encode proteins - *CDH19* at chr18:62,322,301-62,422,196, *DSEL* at chr18:63,324,799-63,334,947, and *TMX3* at chr18:64,491,905-64,533,333. *CCDC102B* was located at the telomeric breakpoint of the deletion at chr18:64,533,471-64,873,406. All of these genes except for *DSEL* have been reported to be located in CNV regions (see Database of Genomic Variants; http://projects.tcag.ca/variation/ [18]–[22]. *TMX3* was located in variation 4061 (chromosome 18: 64,126718-64,543,942) which was duplicated, but not deleted, in 3/270 HapMap individuals [19]. However, CNV studies do not definitively eliminate any of the deleted candidate genes from consideration for the etiology of the microphthalmia, although we assessed *CDH19* as less likely to be involved in the pathogenesis of the eye defects due to the frequency of *CDH19* deletions in normal individuals. We also did not find significant expression of *Cdh19* in the developing murine eye (data not shown).

**Figure 2:**
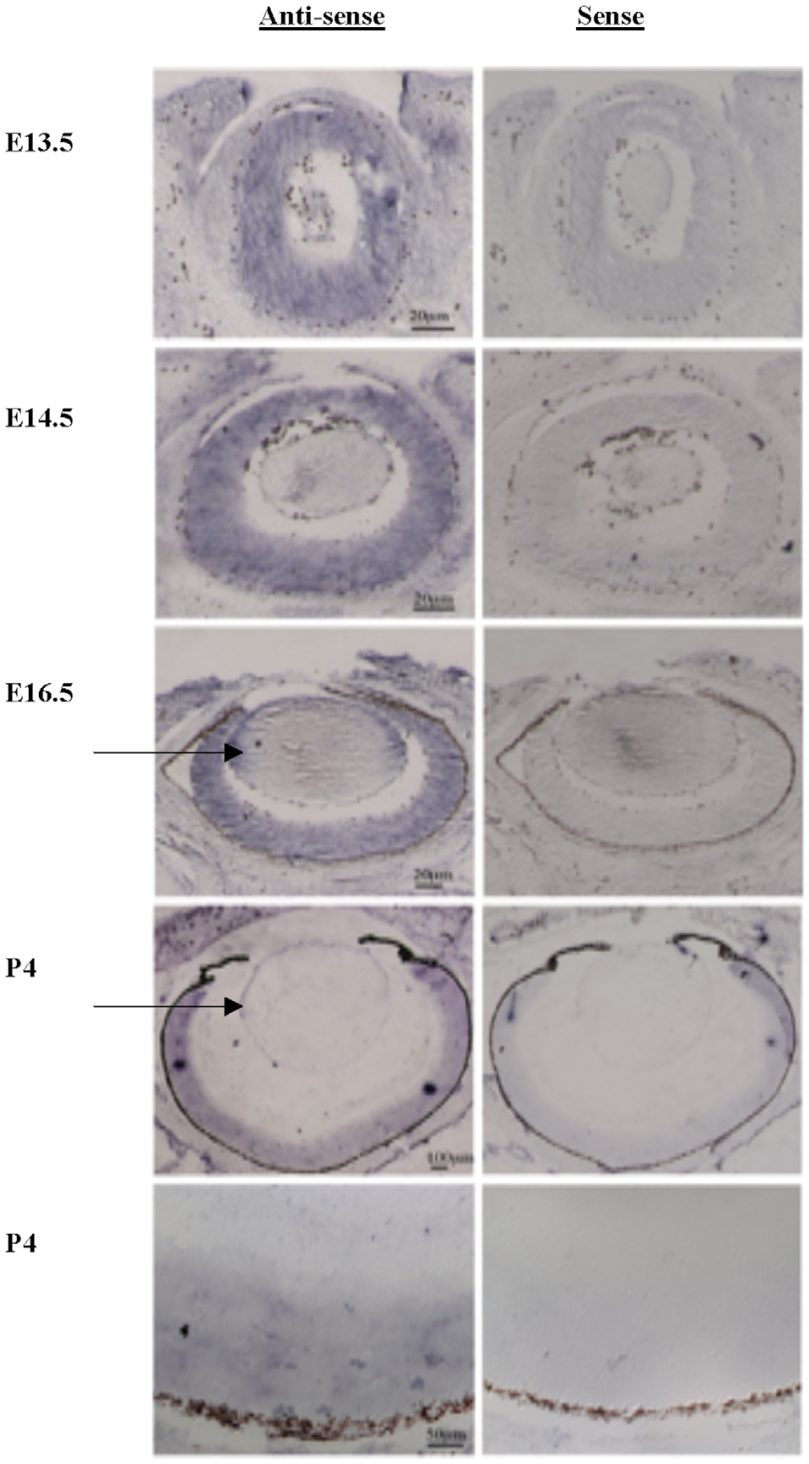
In-situ hybridization shows strong expression of *TMX3* in the developing murine eye. Fig. 2. In-situ hybridization using antisense and sense riboprobes for *TMX3*, showing expression in the murine developing eye at E13.5, E14.5, E16.5 and P4. An arrow points to the labeling of the lens epithelium at E16.5 and P4 with the *TMX3* antisense probe.

There is no murine homologue for *Ccdc102b*, but we did not find significant expression in either developing eye or diaphragm for *Ccdc102a*, the most closely related murine gene (data not shown). *Dsel* was only weakly expressed in the murine lens at embryonic day 13.5 (E13.5; data not shown). In contrast, *Tmx3* expression was first detectable in the developing murine eye at E11.5, but expression was relatively weak (data not shown). From E13.5–E16.5, we saw robust *Tmx3* expression in regions corresponding to the future retina and lens epithelium, two important sites of eye development (Fig. 2). At postnatal day 4 (P4), *Tmx3* expression was present in at least the outer retinal layer of the eye (Fig. 2). In addition to the developing eye, *Tmx3* was also expressed in several other tissues during embryogenesis, including the brain, kidney and lung, confirming the expression data for human *TMX3*, in which 5.1 kb and 4.0 kb transcripts have been found in many tissues such as the eye, heart, skeletal muscle, brain, lung and kidney [23]. Our *in-situ* studies enabled us to prioritize our candidate genes for anophthalmia and microphthalmia, with *Tmx3* becoming the strongest candidate based on the intensity of expression and the duration of expression in the developing eye.

**Figure 3:**
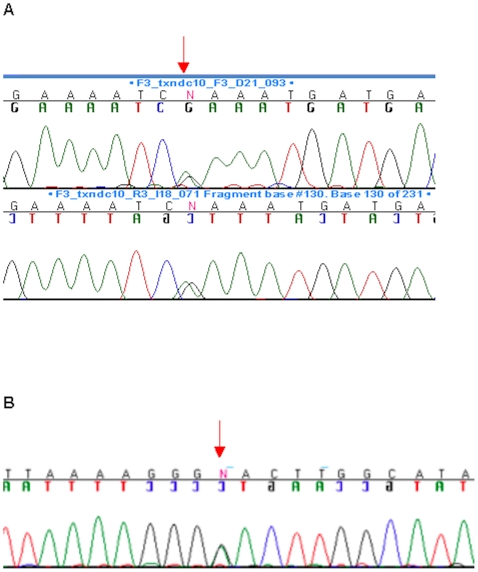
Two patients unrelated to our propositus with microphthalmia have sequence alterations predicting amino acid substitutions in *TMX3*. Fig. 3A. Chromatogram showing c.116G>A, predicting p.Arg39Gln in *TMX3*. Arrow points to the site of the sequence alteration. Fig. 3B. Chromatogram showing c.322G>A, predicting p.Asp108Asn in *TMX3*. Arrow points to the site of the sequence alteration.

Our expression studies lead us to re-sequence *TMX3* in 162 patients with microphthalmia or anophthalmia. We identified a c.116G>A transition, predicting the p.Arg39Gln amino acid substitution, in a Caucasian male who had unilateral microphthalmia and retinal and iris coloboma (Fig. 3A; Table 1; numbering according to transcript ENST00000299608 (Ensembl Database: http://www.ensembl.org/index.html), with 1 = A of ATG start codon). Sadly, parental DNA from this patient was unavailable, and thus it is not known if the sequence alteration occurred *de novo*. This nucleotide change is predicted to substitute a basic residue (arginine) with a polar residue (glutamine) in the catalytic thioredoxin-like domain of the protein [23]. The nucleotide alteration was assessed as ‘possibly damaging’ by the PolyPhen website (http://genetics.bwh.harvard.edu/pph/) for the prediction of functional effect of human non-synonymous SNPs, with a Position-Specific Independent Counts (PSIC) score difference of 1.595. This substitution was not present in 212 Caucasian control chromosomes matching the ethnicity of this Caucasian patient, or in 102 control chromosomes of mixed ethnicity (obtained from the California Birth Defects Monitoring Program), or in 30 control chromosomes of mixed ethnicity (obtained from the Coriell Institute for Medical Research, www.coriell.org/), making a total of 344 control chromosomes without c.116G>A (data not shown). The arginine residue was conserved in multiple species (Table S1), supporting pathogenicity for this sequence alteration.

**Table 1:** Sequence Alterations in *TMX3* in 162 patients with Anophthalmia or Microphthalmia.

Nucleotide^a^	Amino Acid Substitution	dbSNP^b^	Patient Phenotype	Allele Frequency in 162 Sequenced Patients with Anophthalmia/Microphthalmia	Allele Frequency in Control Chromosomes^c^
c.-25G>A (5′UTR)	-	-	Unilateral Microphthalmia, Micrognathia	G = 0.997A = 0.003	Not done
c.116G>A	p.Arg39Gln	-	Unilateral Microphthalmia and Coloboma	G = 0.997A = 0.003	Not detected in 320 Control Chromosomes
c.322G>A	p.Asp108Asn	-	Unilateral Microphthalmia, Micrognathia	G = 0.997A = 0.003	Not detected in 240 Control Chromosomes
c.477A>T	-	-	Bilateral Microphthalmia	A = 0.997T = 0.003	Not detected in 180 Control Chromosomes
c.925G>A	p.Val309Ile	-	Bilateral Microphthalmia	G = 0.988A = 0.012	G = 0.990; A = 0.01 in 188 Caucasian Control Chromosomes
c.997G>A	p.Val333Ile	-	Anophthalmia/Microphthalmia	G = 0.994A = 0.006	Not detected in 188 Caucasian Control Chromosomes. Mother of one patient was Homozygous for A Allele
c.1441A>T (3′UTR)	-	-	Unilateral Microphthalmia with Cyst	A = 0.997T = 0.003	Not done

Nucleotide^a^  =  numbering according to ENST00000299608, with 1 = A of ATG start codon;

dbSNP^b^  =  Database of Single Nucleotide Polymorphisms, http://www.ncbi.nlm.nih.gov/sites/entrez;

Allele Frequency in Control Chromosomes^c^  =  for full details of ethnicity of control chromosomes, please see text.

We also found. c.322G>A, predicting p.Asp108Asn (Fig. 3B; Table 1), also in the thioredoxin-like domain of the protein, in an adopted patient from Haiti who had severe, unilateral left microphthalmia and significant micrognathia (small jaw) but normal development. This substitution was predicted by PolyPhen to be ‘possibly damaging’, with a PSIC score of 1.614, and was also highly conserved in different species (Table S1). Parental samples were unavailable and the ethnicity of the patient was not known, but the substitution was not found in 91 chromosomes of mixed ethnicity (obtained from the Coriell Institute for Medical Research, www.coriell.org/), 48 chromosomes of African American ethnicity, 46 chromosomes of Hispanic ethnicity and 46 chromosomes of Caucasian ethnicity for a total of 231 control chromosomes without c.322G>A (data not shown). Interestingly, this patient also had an alteration of unknown significance, c.-25G>A, in the 5′UTR of *TMX3*, 25 base pairs upstream to the A in the ATG start codon and close to the predicted promoter of the gene (McPromoter – The Markov Chain prediction promoter server; http://tools.igsp.duke.edu/generegulation/McPromoter/). However, we could not obtain RNA to look at *TMX3* gene expression. DNA from this child had previously been sequenced for *SOX2* mutations, and c.859G>C, predicting p.Ala287Pro, was identified (Ms Tanya Bardakjian, personal communication), but this alteration was also of unknown significance, with a PolyPhen PSIC score difference of 1.347, indicating that it could be considered benign. However, in view of this *SOX2* sequence alteration of unknown significance, we chose to focus our attention on p.Arg39Gln rather than p.Asp108Asn for functional studies (see below). We also found numerous other non-coding sequence variants in *TMX3* that have been listed in Table S2.

When assessed by pulse chase experiments, we did not detect any significant difference between wild type *TMX3* and altered *TMX3*(p.Arg39Gln) in terms of protein stability over a time period of 25 hours (data not shown). We therefore sought to make an animal model of *TMX3* deficiency to examine the effect of reduced expression of this gene in eye development in another species and to test p.Arg39Gln for pathogenicity. *Zgc:110025* (Accession number NM_001020557), the *Danio rerio* orthologue of *TMX3*, has 46% identity to human full-length *TMX3* (Clustal W2; http://www.ebi.ac.uk/Tools/) and is single-copy in the zebrafish genome. We designed two antisense morpholinos to reduce the expression of this gene, one directed against the start codon (hereafter referred to as MO1 morpholino, or MO1), and a splice site morpholino (hereafter referred to as MO3 morpholino, or MO3) directed against the exon 2-intron 2 boundary of *zgc:110025*. We verified the ability of the anti-splice morpholino to reduce *zgc:110025* expression using reverse transcription polymerase chain reaction (RT-PCR) at 2 days post fertilization (dpf; Figure S1).

**Figure 4:**
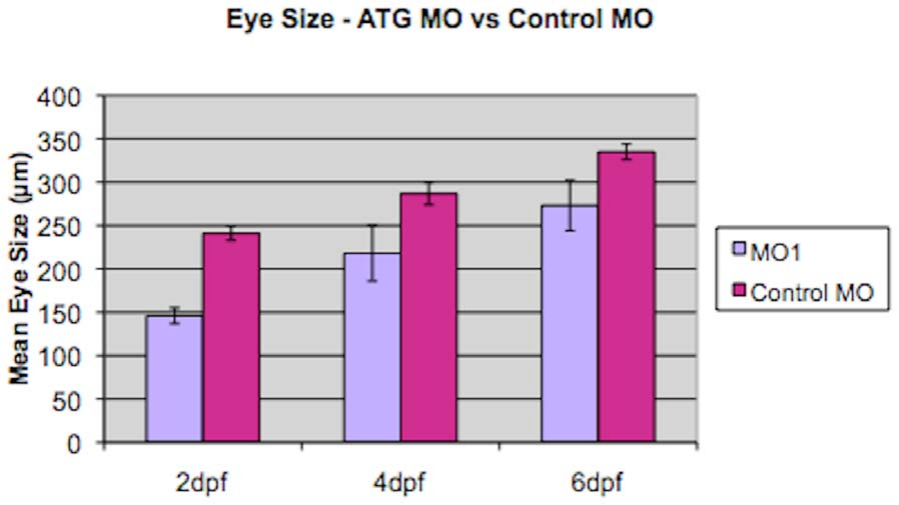
A comparison of eye size in MO1 morpholino-injected larvae and control-injected larvae. Graph shows mean eye size (measured in µm on y-axis) for MO1 morpholino-injected larvae (MO1; light purple) compared to control-injected larvae (Control MO; dark purple) at three different time periods, 2 dpf, 4 dpf and 6 dpf, labeled on the x-axis. Data are shown as mean +/− one standard deviation, measuring a minimum of 9–20 independent retinas per data point. Analysis using a Two-sample t-test assuming equal variance showed a significant difference at 2 dpf, 4 dpf and 6 dpf (p<0.05 for 2 dpf, 4 dpf and 6 dpf).

**Figure 5:**
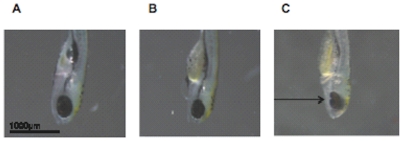
Reduced expression of *zgc:110025* results in a small eye phenotype. Fig. 5A. Control injected, wild type zebrafish showing normal eye formation at 6 dpf. Fig. 5B. MO1 morphant zebrafish showing a small eye compared to control at 6 dpf. Fig. 5C. MO1 morphant zebrafish with co-injection of TMX3/(p.Arg39Gln), showing a small ocular coloboma (indicated by arrow) at 6 dpf. Fish are oriented with ventral surface facing left and dorsal surface facing right.

**Figure 6:**
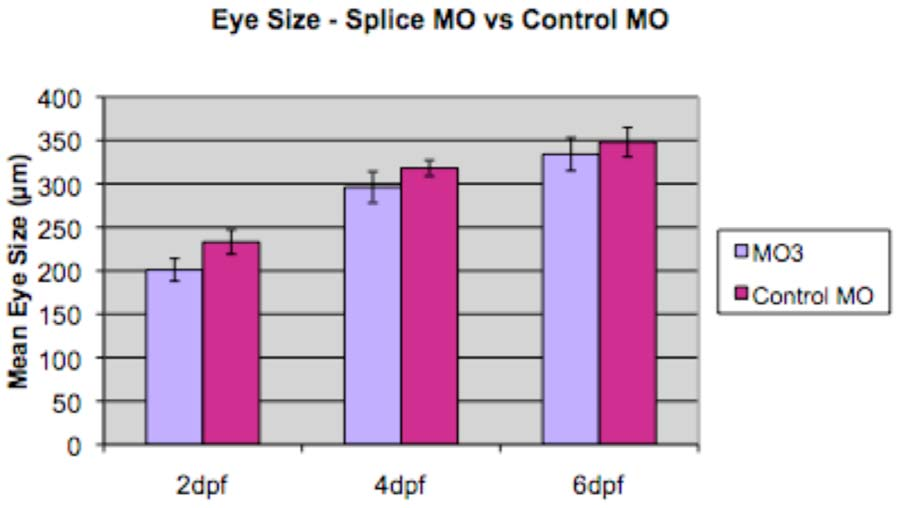
A comparison of eye size in splice morpholino-injected larvae and control-injected larvae. Graph shows mean eye size (measured in µm on y-axis) for splice morpholino-injected larvae (MO3; light purple) compared to control-injected larvae (Control MO; dark purple) at three different time periods, 2 dpf, 4 dpf and 6 dpf, labeled on the x-axis. Data are shown as mean +/− one standard deviation. A representative single experiment is shown, measuring a minimum of 10–20 independent retinas per data point. Analysis using a Two-sample t-test assuming equal variance showed a significant difference at 2 dpf and 4 dpf (p<0.05 for 2 dpf), but not at 6 dpf (p>0.05).

When we injected 6 ng of MO1, there was a significant difference in eye size between larvae injected with MO1 and control-injected larvae at all three time periods examined (p<0.05 for 2 dpf, 4 dpf and 6 dpf; unpaired Two-sample t-test; Fig. 4). At 6 dpf, we also obtained a low, but consistent frequency of coloboma (2.6%; Fig. 5) in morphant larvae that did not have any other evidence of external toxicity. The eye phenotype was commonly bilateral. However, the interpretation of the phenotype obtained by MO1 injection was complicated by a relatively high incidence of toxicity and a wide variation in eye size at 6 dpf (Fig. 4). There was a consistent reduction in eye size for 8 ng of MO3 compared to control-injected larvae at 2 dpf and 4 dpf (p<0.05 for 2 dpf), but not at 6 dpf (Two-sample t-test, Fig. 6). The small eye phenotype was present in the absence of toxicity and was consistent over more than five independent experiments. Coloboma was observed very infrequently in MO3-injected fish, at an estimated frequency of less than 1% (data not shown).

For both morpholinos, we defined penetrance as an eye size greater than 2 standard deviations below the mean eye size for control-injected larvae in the same experiment. Penetrance varied between experiments, but was approximately 80% for microphthalmia in MO1-injected mutants and 50–60% in MO3- injected mutants at 2 dpf. These data using two independent morpholinos indicate that a reduction in expression for the zebrafish orthologue of*TMX3* can be associated with a reduction in eye size.

**Figure 7:**
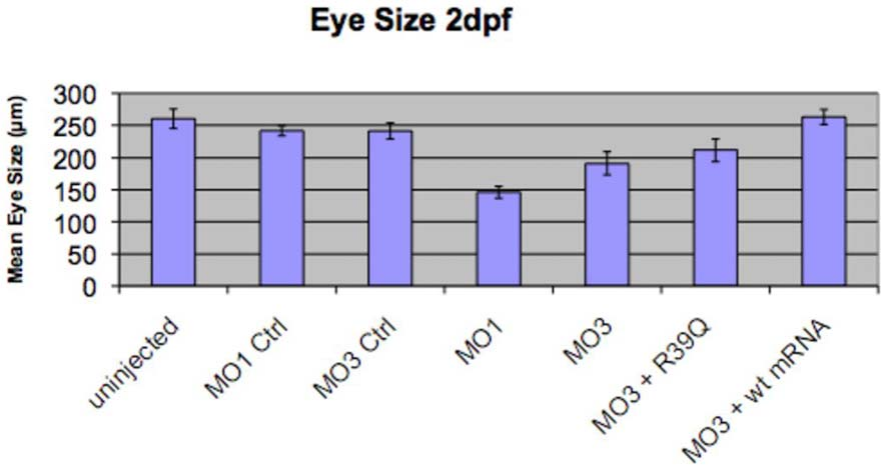
Co-injection of human wildtype *TMX3* mRNA with a splice antisense morpholino rescues morphant eye size. Data are shown for mean eye size (measured in µm on the y-axis) at 2 dpf for uninjected, control-injected, MO1 and MO3 injected, and MO3 and human wildtype and mutant TMX3 mRNA-injected fish (categories listed on x-axis). A minimum of 10–20 fish were scored for each data point and the data is shown as mean +/− one standard deviation. Rescue of the small eye phenotype can be seen by the similarity in eye measurements obtained for MO3 and human wildtype TMX3 mRNA-injected larvae compared to uninjected and control-injected larvae (p>0.05; two-sample t-test assuming equal variance).

We tested for rescue of the MO1 and MO3 eye phenotype using human wildtype mRNA for *TMX3*. In preliminary experiments, injections of 100–120 pg of human wildtype mRNA for *TMX3* did not result in an external phenotype different from uninjected or control-injected fish (data not shown). Injection of 100–120 pg of human *TMX3* wild type mRNA together with MO1 or MO3 dramatically reduced the frequency of ocular abnormalities compared to experiments with injections of MO1 or MO3 alone at 2 dpf (Table 2; Fig. 7), indicating that rescue of the external ocular phenotype had occurred. This experiment also established the specificity of our MO1. However, the injection of 100–120 pg of human *TMX3*(p.Arg39Gln) mutant mRNA, analogous to the human mutation, together with MO1 morpholino did not rescue the small eye phenotype (Table 2). In the dual injection rescue experiments with MO1, we did observe a high frequency of external toxic effects with the combined morpholino and mRNA injections that may in part have been attributable to early gene expression. In our dual injection rescue experiments with MO3 and wildtype human *TMX3* mRNA, rescue occurred (Fig. 7), but minimal toxicity was observed (data not shown). Human *TMX3*(p.Arg39Gln) mutant mRNA did not rescue the MO3 phenotype (Fig. 7).

**Table 2:** Frequency of Microphthalmia and/or Coloboma with 6 ng MO1 Morpholino and 100 pg human *TMX3* wild type or 100 pg of human *TMX3*/(p.Arg39Gln).

	MO1 Morpholino (n = 349)	MO1 Morpholino +100 pg Human TMX3 wild type (n = 582)	MO1 Morpholino +100 pg Human TMX3/(p.Arg39Gln) (n = 440)
**Microphthalmia/Coloboma^a^**	18 (5.2%)	7 (1.2%)^c^	30 (6.8%)^d^
**External Toxicity^b^**	46 (13.2%)	164 (28.18%)	77 (17.5%)
**Normal**	285 (81.6%)	411 (70.62%)	333 (75.7%)

Microphthalmia/Coloboma^a^: Larvae with microphthalmia or coloboma only and without any other evidence external toxicity.

External toxicity^b^: Edema, body axis curving; can also include eye defects.

c : P = 0.0031 for the frequency of microphthalmia and/or coloboma using MO1 morpholino versus frequency of microphthalmia and/or coloboma using MO1 morpholino +100 pg Human *TMX3* wild type. Fish with external toxicity were excluded from the analysis. A two-tailed Fisher's exact test was used.

d : P = 0.293 for the frequency of microphthalmia and/or coloboma using MO1 morpholino versus frequency of microphthalmia and/or coloboma using MO1 morpholino +100 pg Human *TMX3*(p.Arg39Gln). Fish with external toxicity were excluded from the analysis. A two-tailed Fisher's exact test was used.

**Figure 8:**
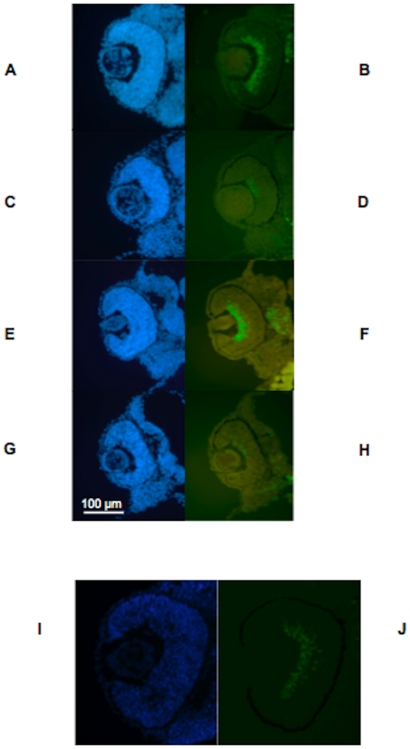
Islet-1 expression is reduced in morphant larvae compared to control larvae at 2 dpf. Fig. 8A–D. Fig. 8A–B. Labeling of MO1 control-injected zebrafish eye with DAPI (Fig. 8A) and FITC (Fig. 8B) showing labeling for islet-1 at 2 dpf. Fig. 8C–D. Labeling of MO1-injected zebrafish eye with DAPI (Fig. 8C) and FITC (Fig. 8D) showing almost absent labeling for islet-1 at 2 pdf. Fig. 8E–H. Fig. 8E–F. Labeling of MO3 control-injected zebrafish eye with DAPI (Fig. 8E) and FITC (Fig. 8F) showing labeling for islet-1 at 2 pdf. Fig. 8G–H. Labeling of MO3-injected zebrafish eye with DAPI (Fig. 8G) and FITC (Fig. 8H) showing almost absent labeling for islet-1 at 2 pdf, similar to the labeling pattern observed with MO1. Fig. 8I–J. Fig. 8I. MO3-injected zebrafish rescued with human wildtype *TMX3* mRNA labeled with DAPI (Fig. 8I) and FITC (Fig. 8J) at 2 dpf, showing labeling with islet-1 antibodies that is similar to labeling in control-injected fish. Ventral is down in all image panels.

The difference in eye size between MO3 and control was evident at 2 dpf, and we therefore chose islet-1 antibody to label retinal ganglion cells and primary neurons. Islet-1 antibody is directed against a LIM homeodomain protein and labels both retinal ganglion cells and other primary neurons. Islet-1 is expressed in the ventral retina at 33 hours post fertilization (hpf [24]). Labeling with islet-1 antibody on sections from MO1 and MO3 fish at 2 dpf showed almost absent expression compared to control-injected fish (Fig. 8C–D and Fig. 8G–H). The labeling difference using islet-1 antibody was present in MO1 morphant larvae at day 2, but was no longer apparent using either morpholino by 4 dpf (data not shown). Co-injection of human wildtype mRNA with MO3 rescued the reduction in islet-1 labeling (Fig. 8I–J), again demonstrating the specificity of the morpholino.

**Figure 9:**
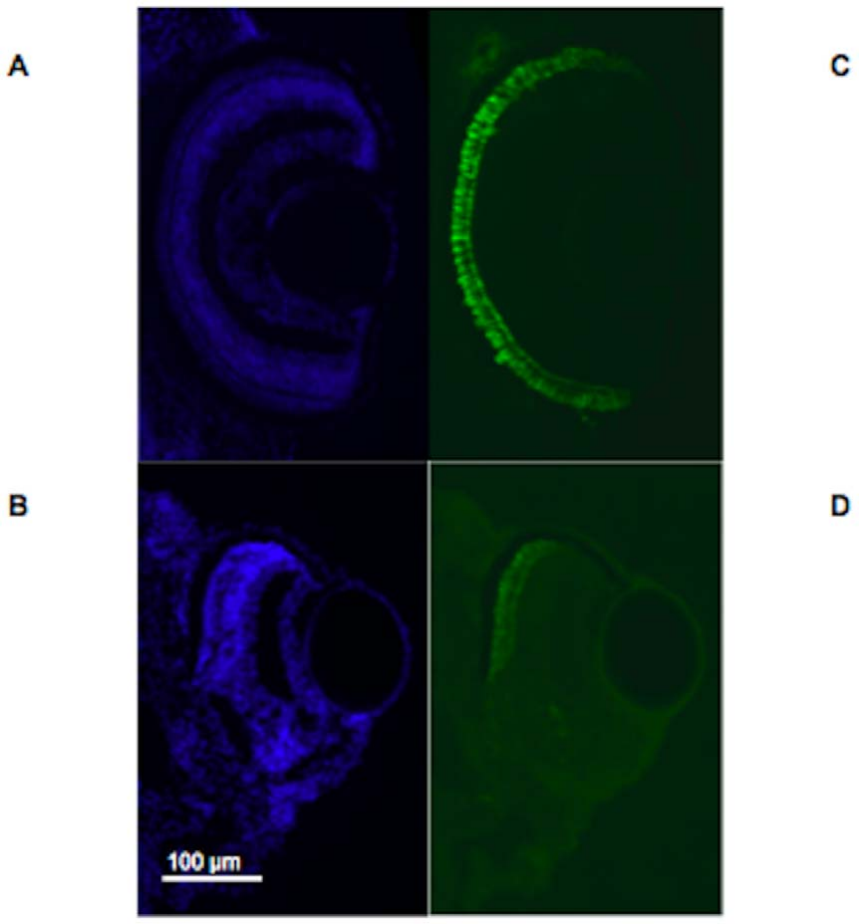
Morphant larvae show altered formation of the ventral eye. Fig. 9A–D. Fig. 9A–B. Staining of control injected zebrafish eye with DAPI and FITC at 6 dpf to image zpr-1 shows a strong signal that can be seen at the ciliary margins of the retina. Fig. 9C–D. Staining of anti-ATG morphant (MO1) zebrafish eye with DAPI and FITC at 6 dpf to image zpr-1 shows absent signal for zpr-1 at the ventral region of the retina in an eye with a coloboma, whereas staining at the dorsal region of the retina appears normal. Fish are oriented so that the ventral surface of the eye is seen inferiorly in each photograph.

On sections from 4 dpf embryos injected with MO1 or MO3 or controls, we measured retinal circumference and the length of antibody labeling for antibodies directed against PKCα, parvalbumin and glutamine synthetase. We chose 4 dpf, as labels for these later developing cell types were not always visible at 2 dpf. We found no significant difference in labeling length when both morpholinos were examined for antibodies against PKCα, parvalbumin and glutamine synthetase (data not shown), labeling for bipolar, amacrine and Mueller glial cells respectively. We also found no significant differences for MO1 for zpr-1 labeling where the retina was intact (Fig. 9A–B), anti-rhodopsin antibody, lectin to Peanut Agglutinin (PNA) and opsin antibodies to label blue and UV cones at 6 dpf (data not shown). We did not examine the rod and cone labels in MO3 larvae at 6 dpf, as the difference in eye size had resolved by this time period.

Finally, we used anti-histone H3 and Terminal deoxynucleotidyl transferase dUTP nick end labeling (TUNEL) to examine cell proliferation and apoptosis in the MO1-injected morphant retinas. Although we found a significant increase in anti-histone H3 labeling at the retinal margins in the MO1 morphants at both 4 and 6 dpf, we did not observe this significant difference for MO3-injected larvae (data not shown). We did not find evidence of increased apoptosis in the MO1-injected fish as compared to controls (data not shown).

**Figure 10:**
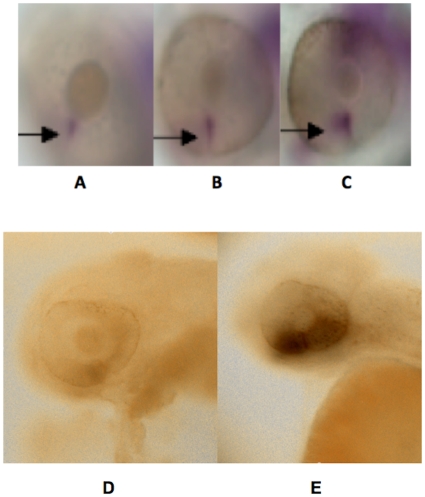
*Pax2* and *Vax2* expression are dysregulated in the region of the choroid fissure in MO1 injected morphant larvae. Fig. 10. Fig. 10A–C. Representative, uninjected (Fig. 10A), control morpholino injected (Fig. 10B) and MO1 morpholino injected (Fig. 10C) whole embryos hybridized with a *Pax2* probe. The region of *Pax2* labeling at the site of the choroid fissure (indicated by arrows) is enlarged in the MO1 morphant embryo compared to both the uninjected and control-injected embryos. Fig. 10D. Representative control morpholino injected (Fig. 10D–E) and splice morpholino (MO3) injected (Fig. 10E) whole embryos hybridized with a *Vax2* probe. The region of *Vax2* labeling (indicated by arrows) is enlarged in the MO3 morphant embryo compared to the control-injected embryo.

A small proportion of MO1- and MO3-injected morphant larvae were observed to have colobomas at the site of the choroid fissure. As colobomas have been previously associated with abnormal *Pax2* expression, we hypothesized that *Pax2* expression could be dysregulated in the region of the choroid fissure in the morphant larvae. We examined *Pax2* expression in whole embryos by *in-situ* hybridization at 2 dpf to determine if expression of this gene was altered between morphants and control-injected larvae. We chose this early time period in order to assess *Pax2* expression at the time of choroid fissure fusion. We found that there was an expansion of *Pax2* expression in the region of the ventral retina and choroid fissure in the morphant embryos compared to control-injected embryos (Fig. 10A–C), suggestive of either increased *Pax2* expression or a delay in the loss of *Pax2* expression due to later optic fissure closure. We also examined *Vax2* expression in MO3-injected eyes at 2 dpf due to the known role of this gene in ventral eye formation and detected increased labeling in morphants compared to control-injected larvae (Fig. 10D–E).

## Discussion

We have previously identified a patient with microphthalmia and a 2.7 Mb deletion at 18q22.1. Our data support a role for one of the deleted genes, *TMX3*, in eye development, as deficiency of this gene in humans and in zebrafish causes microphthalmia. Our forward genetic approach, starting with the human birth defect of microphthalmia, has resulted in the discovery of an important function in eye development for a vertebrate gene.

*TMX3* is a member of the protein disulfide isomerase (PDI) family and contains a thioredoxin domain that catalyzes the formation of disulfide bonds [23], [25]. Members of the thioredoxin superfamily share two features – a short sequence motif, CXXC, that represents the active site for the oxido-reductase reaction, and an overall structure containing this motif that forms a thioredoxin-like fold. Human *TMX3* has 16 exons that encode a 454 amino acid, 51.8 kDa protein that is a single pass membrane protein of the endoplasmic reticulum (ER [23]). The protein has two isoforms and is N-glycosylated. The luminal region of *TMX3* has an N-terminal ER signal peptide (residues 1–24), a catalytic thioredoxin-like domain that contains the active CGHC sequence as described above (residues 25–131), two redox-inactive thioredoxin-like domains, a transmembrane domain and a C-terminal domain with a KKXX motif [23], . The overall sequence similarity is 93% for murine *TMX3* and 28% for the *D. melanogaster* and *C.elegans* orthologous proteins.

There is prior evidence that thioredoxin genes may play a role in eye formation. In Drosophila, the drosopterins, or red components of the eye, are partially encoded by the *clot* gene, a member of the Glutaredoxin class of the thioredoxin-like enzyme superfamily [26]. Disruption of murine *TMX3*, *Rttn, CD226* and *Dok6* genes with a biallelic, 1.6 Mb deletion causes the lethal Nt (no turning) phenotype in mutant mice [27], [28]. Although the phenotype of the Nt mouse is caused by *Rttn* deficiency, the breakpoint of the Nt deletion interrupts *TMX3*, which shows no transcript. Mutant Nt embryos have otic eminences but lack optic eminences at E9.5 [27]. However, the mice are only viable until E11.5, and thus further study of eye development in these mice is not feasible.

Our *in-situ* hybridization experiments showed that *TMX3* was expressed in the murine eye from E11.5 onwards, with strongest expression in the retinal neuroepithelium from E13.5 to E16.5 (Fig. 2). The gene is more widely expressed at later stages, with labeling in the brain, lungs, kidneys and spinal ganglia. In contrast, the zebrafish orthologue of *TMX3*, *zgc:110025*, is initially expressed in the hypophysis of the developing brain and in the retina, and subsequently in the photoreceptor layer of the retina from 30 hours post fertilization until at least day 5 of development (Thisse B, Thisse C, The Zebrafish Model Organism Database (ZFIN) Direct Data Submission: http://zfin.org [29]). The early expression data are consistent with our morpholino results, which show the greatest difference in eye size at 2 and 4 dpf (Fig. 4 and Fig. 6). However, the expression pattern is more restricted in fish than in mouse. If one assumes that a similar expression pattern in humans and mice is likely, the eye phenotype in patients is very specific and is so far unilateral, despite the apparently widespread expression of this gene in mammals. This finding could be due to an intrinsic sensitivity of the process of eye formation to stressors, meaning that a hypomorphic allele may be more penetrant in the eye than in other body organs [30].

Both of our morpholinos resulted in small eyes with a low frequency of coloboma, reduced islet-1 labeling and both were rescued by human wildtype mRNA, demonstrating the specificity of the morpholinos for *TMX3*. In addition, human wildtype *TMX3* mRNA was able to rescue the severe reduction in islet-1 labeling exhibited in morphant eyes (Fig. 8I–J). However, there were some differences in phenotype between MO1 and MO3-injected morphant eyes, possibly resulting from greater gene knockdown with MO1 or a longer duration of knock-down, although we were unable to perform a Western blot to directly verify the level of gene reduction with MO1 because of lack of a suitable antibody.

We noted that some of the morphant eyes had incomplete formation of the lens (data not shown). We chose to concentrate on the retinal phenotype in the morphants, and have not studied lens formation in the morphants, despite the expression of *TMX3* in the lens epithelium (Fig. 2), but it is possible that there is also a contribution to the microphthalmia from abnormal lens formation in the morphants. Interestingly, the microphthalmia phenotype was unilateral in all of the three human patients with *TMX3* sequence alterations, whereas we found a bilateral reduction in eye size in fish. Although this is a small sample size, we postulate that the *TMX3* phenotype shows extreme variation in the resultant degree of delay in eye formation, and that reduced *TMX3* gene dosage can be compensated for by different genes. Many of the genes that cause anophthalmia, microphthalma and coloboma have been associated with marked phenotypic variation or an inconsistent eye phenotype, both for the spectrum of eye defects and in terms of associated anomalies [31].

We found coloboma, or failure of the choroid fissure to close, in addition to microphthalmia, in the eyes of the morphant larvae injected with MO1 and MO3, although the frequency of coloboma was very low (MO1 frequency of coloboma of 2.6% from three independent experiments; MO3 frequency of coloboma <1% from five independent experiments). Ocular coloboma is a ventral patterning defect has been shown to be associated with abnormal *Pax2* and *Vax* expression [32]. We hypothesized that *Pax2* expression could be dysregulated in the region of the choroid fissure in morphant larvae, and our whole embryo studies showed increased labeling of *Pax2* in morphants compared to controls at 2 dpf. At first, this result appeared contradictory, as *Pax2* loss of function and/or haploinsufficiency, rather than a gain of function, has been associated with the formation of colobomas, microphthalmia and optic nerve defects in both humans and animals [33], [34]. *Pax2* is expressed in the central optic cup at the time of choroid fissure closure, when it becomes restricted to the cells of the optic stalk and the cells that line the choroid fissure to regulate the breakdown of the extracellular matrix lining the choroid fissure [32]. Lack of *Pax2* expression results in persistence of the basal lamina and matrix at the sides of the choroid fissure, preventing the fusion of the neuroepithelium and causing coloboma [35]. However, it has also been shown that ectopic expression of *Pax2* in the ventral optic cup after electroporation in the chick can leads to a failure of choroid fissure closure, phenocopying the colobomas seen with *Pax2* loss of function [32]. The increased labeling seen with the *Pax2* and *Vax2* probes could also indicate a delay in the closure of the optic fissure in morphants and the formation of the ventral eye, as *Vax2* expression is present in the optic cup, optic stalk and presumptive neural retina from 24–30 hpf in wildtype fish (The Zebrafish Model Organism Database (ZFIN) Direct Data Submission: http://zfin.org).

Based on our data, we propose that deficiency for *TMX3* causes a small eye phenotype and may be involved in the pathogenesis of human microphthalmia in three known cases. The propositus most likely had a loss of function mutation because his deletion involved the entire gene, but the effects of the two missense substitutions have not been quantified in terms of loss or gain of function. Reduced penetrance between individuals (the patient inherited the deletion from his asymptomatic mother), and within individuals (all three patients described in this study had unilateral rather than bilateral microphthalmia) suggests that haploinsufficiency can reduce *TMX3* expression to levels near a pathogenic threshold. Other genetic modifier effects and environmental factors that influence *TMX3* levels relative to this threshold are likely to be responsible for determining the penetrance of the eye defects. Our rescue data using the wildtype human mRNA to prevent a morpholino-induced reduction in eye size argue strongly that gene deficiency is required for the phenotype.

We have examined the genes in a 2.7 Mb deletion at chromosome 18q22.1 in a male with microphthalmia for a role in the pathogenesis of microphthalmia and present evidence that one of the deleted genes, *TMX3*, is involved in eye development. We noted strong expression of *TMX3* in the murine eye from E13.5 onwards that persisted into postnatal life. We identified two sequence alterations that predict amino acid substitutions, c.116G>A, predicting p.Arg39Gln, in an unrelated patient with unilateral microphthalmia and coloboma, and c.322 G>A, predicting p.Asp108Asn, in a female with unilateral microphthlamia and severe micrognathia. We showed that a reduction in the expression of the orthologous gene in zebrafish, *zgc:110025*, using two antisense morpholinos, resulted in an external phenotype of small eye and in abnormal retinal formation, with reduced labeling with islet-1 antibody at 2 dpf. Finally, increased *Pax2* and *Vax2* labeling at the site of the choroid fissure in the morphant-injected embryos compared to control-injected embryos suggests delayed closure of the optic fissure and may implicate these genes and/or the *Pax2* pathway in the pathogenesis of the colobomas. We hypothesize that a deficiency of *TMX3* predisposes to microphthalmia, and that further study of this gene and other thioredoxins in eye development is warranted.

## Methods

All patient samples were collected after ethical approval by an Institutional Review Board at UCSF and at all institutions where participants were recruited. At UCSF, we obtained human samples after informed, written consent from participants or their parents/guardians under a protocol approved by the Committee for Human Research (CHR) at the University of California, San Francisco (CHR number 41842-22157-06). DNA samples from a further 24 patients with anophthalmia and/or microphthalmia were collected from Tanya Bardakjian, CGC and Dr Adele Schneider under an approved protocol for the Anophthalmia/Microphthalmia Registry and gene screening project (Institutional Review Board, Albert Einstein Medical Center). DNA samples from 66 patients with anophthalmia/microphthalmia were obtained from Dr. David FitzPatrick, MRC Human Genetics Unit, Edinburgh with approval from the Lothian Research Ethics Committee. DNA samples from 36 patients with unilateral or bilateral microphthalmia collected under institutional review board approved protocols were also obtained from Pr. Patrick Calvas, Inserm U563 (Review Board: CPP Sud Ouest et Outre Mer II) and from Dr Daniel Schorderet (Institute Review Board of the Institut de Recherche en Ophtalmologie) respectively. All zebrafish work was done in accordance with an Institutional Animal Care and Use Committee (IACUC) approved protocol to Dr Herwig Baier at the University of California, San Francisco.

DNA was extracted from peripheral blood lymphocytes and cell lines using proteinase K and salting-out according to standard methods (Qiagen, Valencia, CA). Phenotypic details were obtained from referring clinicians.

Array hybridization was performed with the GeneChip® Human Mapping 500K Set to fine map the deletion. Hybridization was preformed using 500 ng genomic DNA according to the manufacturer's instructions. The results were analyzed according to the Significance of Mean Difference (SMD) algorithm designed to detect copy number variations [36] and parental studies using the same mapping set were performed.

Section *in-situ* hybridization on murine paraffin- or cryo-sections was performed as previously described [37] using digoxygenin-labeled riboprobes (DIG RNA labeling kit; Roche, Indianapolis, IN). The *TMX3* sense and antisense probes (1444 bp) were generated using the full-length cDNA clone (MMM1013-9200944, Open Biosystems, Huntsville, AL). *Dsel* and *Ccdc102a* probes were generated using the following primers: *Dsel*: F: 5′GAGTGAGTGCGTGTGTCCAG; R: 5′ TCTCGTTTTTGTGTGCAAGG; *Ccdc102a*: F: 5′AGCCATCTTTCGCTGTCTGT; R: 3′TGTTCCATCTCTGCACGAAG. *Dsel*, *TMX3 and Ccdc102a* expression were examined in the murine eye at E11.5, E12.5 and E13.5. *TMX3* expression was also examined in the murine eye at E14.5, E16.5, E18.5, P1 and P4, to sample time-points from both embryonic and postnatal development.

Genomic sequencing was performed with a BigDye® Terminator v3.1 Cycle Sequencing Kit on an ABI 3730 machine (Applied Biosystems, Foster City, CA) as previously described [14]. We sequenced the coding exons and intron-exon boundaries of *TMX3* in DNA samples from 162 patients with anophthalmia or microphthalmia.

Fish were maintained and bred under standard conditions at 28°C, and embryos were staged according to dpf. Anti-sense morpholinos targeting the MO1 start codon (ATG; −6 to +19; TGTTTCTCATGTTTGCCATCTTGAG; referred to as MO1 morpholino or MO1 in text), and the splice donor site between exon 2 and intron 2 (GTAAAATACTTAC-TTGTCATCGAGC; referred to as splice morpholino or MO3 in text) of *zgc:110025* were obtained (GeneTools LLC, Philomath, OR). A BLAT search for both MO1 and MO3 did not reveal any similarity to other sequences that might result in off-target effects. We also obtained a specific control for the MO1 morpholino above (TGATTCTGATGTTTCCCATGTTCAG), a specific control for the splice morpholino (GTATAATAGTTAGTTGTGATCCAGC) and a standard control (GeneTools LLC, Philomath, OR). We injected 2–8 ng of antisense morpholino or control morpholino into eggs at the 1–8 cell stage and examined larvae at 2 dpf, 4 dpf and 6 dpf for external eye defects and for signs of toxicity. Eye size (measurement of longest eye axis) was measured in µm using direct microscopy (AxioVision Digital Imaging Software, Carl Zeiss Microimaging Inc., Thornwood, NY) using a micrometer for standardization. A minimum of 10 to 20 fish were measured for each data point in independent experiments for all data points, except for MO1 at 6 dpf, when 9 fish were measured. We did not observe a significant phenotype with either the standard or the specific control morpholinos.

RNA was obtained using Trizol and cDNA was prepared using the High-Capacity cDNA Reverse Transcription kit (Applied Biosystems, Foster City, CA). RT-PCR to verify gene knock-down for the splice morpholino was performed using forward primer TTACGCGGTCAATGACAAGA from exon 1 of *zgc:110025* and reverse primer CTCCACCAGCCAGAGTTCAT from exon 3 of *zgc:110025* (data not shown). The cDNA was also amplified using universal primers for actin used to check for cDNA integrity. For the MO1 morpholino, we ran a Western blot using the only available antibody for *TMX3* [23] and were unable to see signal from protein extracted from 2 dpf larvae from either MO1 morpholino larvae or control morpholino larvae, although an antibody for actin verified the integrity of the protein samples (data not shown).

We used pcDNA3/*TMX3*-HA and pcDNA3/*TMX3*(p.Arg39Gln)-HA [23] as template to make mRNA. The C-terminal end of the protein was altered to ASYPYDVPDYASKKKD from human wild type PTVQEPKDVLEKKKD [23], resulting in loss of the stop codon that could theoretically reduce translation or message stability [38], but both constructs have been previously used to examine gene function [23]. Human *TMX3* wild type mRNA and human *TMX3*(p.Arg39Gln) mRNA were made by digestion with ApaI and sense RNA was transcribed, capped and a polyA tail added using the mMessage mMachine® T7 Ultra kit (Ambion, Austin, TX) according to manufacturer's instructions. Constructs were sequenced to verify the addition of a polyA tail (data not shown). We initially injected 100–120 pg of human *TMX3* wild type mRNA into wild-type zebrafish eggs to ensure that there was no detectable phenotype with overexpression of the gene, and we did not detect any increase in external ocular abnormalities beyond control frequencies at this dose (data not shown). We then performed a series of experiments injecting 6 ng of MO1 morpholino or 8 ng of MO3 morpholino with either 100–120 pg of human *TMX3* wild type mRNA or 100–120 pg of *TMX3*(p.Arg39Gln) mRNA in a total injection volume of 1 nl, similar to other injection volumes.

Fixed larvae were cryoprotected in 35% sucrose for a minimum of 4 hours. The larvae were embedded in cryomatrix (Tissue Freezing Medium, Triangle Biomedical Sciences, Durham, NC) and rapidly frozen on dry ice. 12 µm sections were cut at −20°C onto slides (VWR Superfrost Plus, West Chester, PA) and air-dried. For immunohistochemistry, slides were washed in phosphate buffered saline (PBS), blocked in PBS containing 3% sheep serum and 0.3% Triton-X and then incubated with primary antibody at 1∶100 to 1∶400 dilution overnight at 4°C. After washing with PBS, slides were incubated with fluorescently labeled secondary antibodies conjugated to AlexaFluor 488 (Invitrogen, Carlsbad, CA) at a 1 in 200 to 1 in 500 dilution for 2 hours at room temperature.

The primary antibody used for 2 dpf larvae was murine monoclonal islet-1 antibody [24]. The primary antibodies used for 4 dpf larvae were rabbit anti-histone H3 (Upstate Biotechnologies/Millipore, Billerica, MA) to label proliferating cells, murine anti-glutamine synthetase (Chemicon/Millipore, Billerica, MA) to label Mueller glial cells, rabbit anti-PKCAα (Santa Cruz Biotechnology Inc, Santa Cruz, CA) to label bipolar cells and murine anti-parvalbumin (Chemicon/Millipore, Billerica, MA) to label amacrine cells. The primary antibodies used to examine photoreceptors at 6 dpf were: murine zpr-1 (Zebrafish International Resource Center, Eugene, OR) for labeling of double cone photoreceptors [39], murine anti-rhodopsin antibody (0.5 mg/ml; Meridien Life Sciences, Cincinnati OH) for labeling of the rod photoreceptor cells and peanut agglutinin (PNA) lectin directly conjugated to TRITC (TRITC Arachis hypogaea lectin, EY laboratories, San Mateo, CA) as a label for the outer segments of the long-double and long-single cone photoreceptors. We also used rabbit anti-blue opsin and rabbit anti-UV opsin kindly provided by Dr David Hyde of the University of Notre Dame according to published protocols [40]. A minimum of 8 independent retinas were examined for each antibody.

Slides were photographed using a Leica fluorescent microscope and SPOT imaging software, or confocal microscopy as described below. Quantification of labeling differences for anti-histone H3 was performed by counting the number of FITC-fluorescent cells in a minimum of two to three, non-adjacent sections per retina for 8 retinas for both MO1 morphant and control-injected larvae. The length of positive, FITC-labeled retina was measured for both MO1 morphant and control injected larvae. Retinal circumference was also measured for the same retinas to control for eye size. A similar methodology was employed for the glutamine synthetase, parvalbumin and PKCα antibodies, but 4-6 independent retinas were scored. Labeling widths were initially compared using a Two-sample t-test assuming equal variance. In instances where a significant difference was found with this statistical method, the analysis was repeated with regression analysis or ANOVA to control for retinal size including the measurements of retinal circumference.

Cell death was detected using the TUNEL assay (terminal deoxynucleotidyl transferase [TdT]-mediated deoxyuridinetriphosphate [dUTP] nick end-labeling method (Apoptag Peroxidase *In-situ* Apoptosis Detection kit, Chemicon, Temecula, CA), according to the manufacturer's instructions on sections from 4 dpf.

*In-situ* hybridization on whole morphant and control larvae from 2 dpf was performed using a probe for the zebrafsh *Pax2* and *Vax2* genes according to standard methodologies [39]. Labeled areas were quantified on images in Photoshop using ImageJ software. For the *Pax2* hybridizations, 13 morphants and 11 control-injected larvae were examined. For the *Vax2* hybridizations, 22 morphants, 15 control-injected and 21 uninjected larvae were examined.

Confocal images were captured using the Zeiss LSM 5 Pascal microscope and software under a 40X oil immersion lens. Confocal stacks were further processed using ImageJ software. In some cases, z-projections of a few slices were made; in others, single, representative slices were selected. In all figures, comparisons were made between images that were processed equivalently—slices compared to slices, and projections compared to projections of a similar number of slices.

## Supporting Information

Figure S1 RT-PCR at 2 days post fertilization shows the expected 154 bp band in lane C, containing cDNA from uninjected larvae, and a very faint 154 bp band in lane MO3, containing cDNA from MO3 injected larvae, with probable RNAi mediated decay of the expected mutant 110 bp splice band in the MO3 lane. The primers used are provided in the text of the paper and the 100 base pair (bp) and 200 bp bands from the size marker are indicated.
(0.47 MB TIF)

Table S1 Conservation of amino acids p.39Arg and p.108Asp between different species for the *TMX3* gene and orthologues.
(0.18 MB DOC)

Table S2 Non-coding sequence alterations in *TMX3* in 162 patients with Anophthalmia/Microphthalmia.
(0.05 MB DOC)
