# Supplementary figures and images for: A Male with Unilateral Microphthalmia Reveals a Role for TMX3 in Eye Development
Source: PLoS One. 2010 May 11;5(5):e10565. doi: 10.1371/journal.pone.0010565 (PMC2868029; doi:10.1371/journal.pone.0010565)

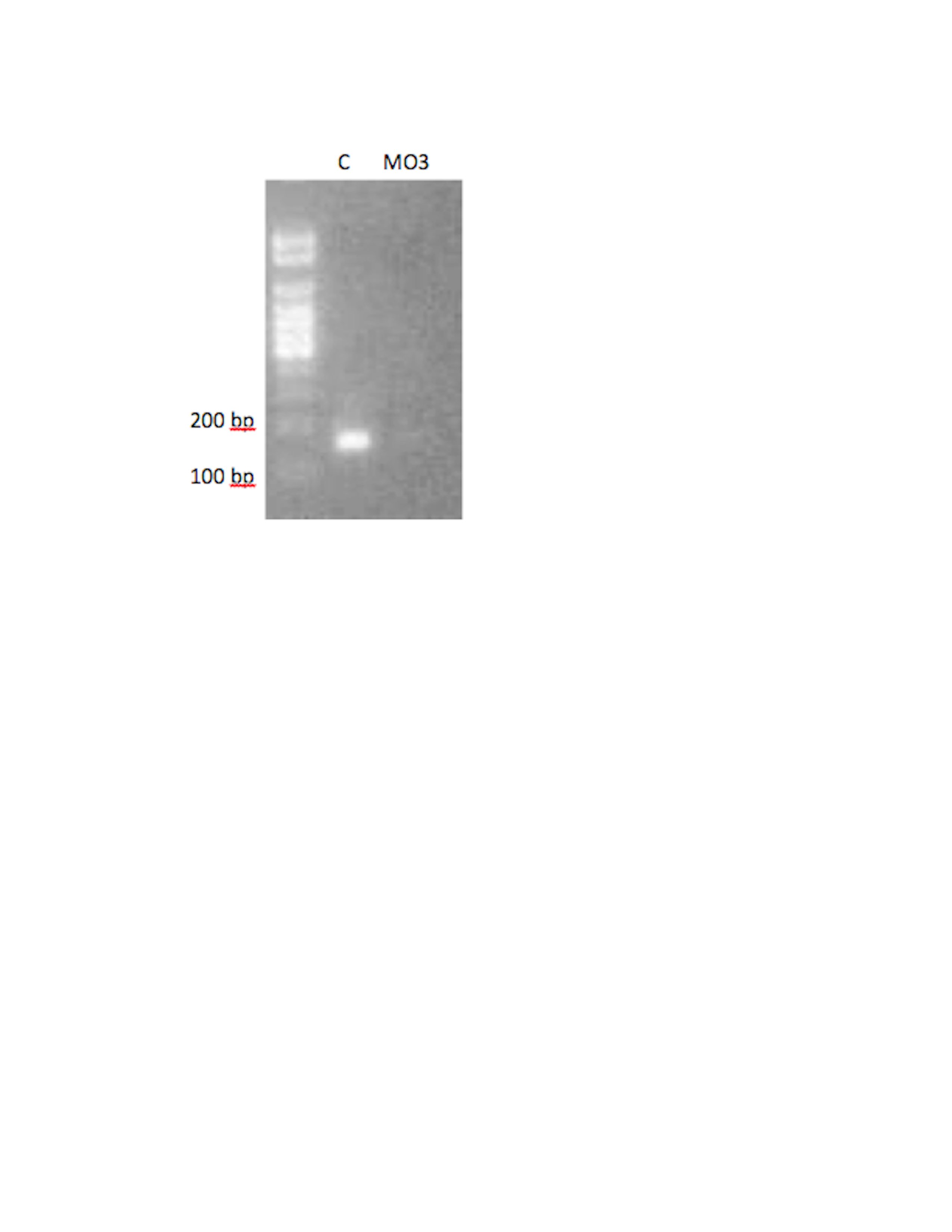

Supplement: Figure S1 — RT-PCR at 2 days post fertilization shows the expected 154 bp band in lane C, containing cDNA from uninjected larvae, and a very faint 154 bp band in lane MO3, containing cDNA from MO3 injected larvae, with probable RNAi mediated decay of the expected mutant 110 bp splice band in the MO3 lane. The primers used are provided in the text of the paper and the 100 base pair (bp) and 200 bp bands from the size marker are indicated. (0.47 MB TIF) [file pone.0010565.s001.tif]
