# Supplementary material for: A Male with Unilateral Microphthalmia Reveals a Role for TMX3 in Eye Development
Source: PLoS One. 2010 May 11;5(5):e10565. doi: 10.1371/journal.pone.0010565 (PMC2868029; doi:10.1371/journal.pone.0010565)
Supplement: Table S1 — Conservation of amino acids p.39Arg and p.108Asp between different species for the TMX3 gene and orthologues. (0.18 MB DOC) [file pone.0010565.s002.doc]

**Table S1. Conservation of Amino Acids p.39Arg and p.108Asp Between Different Species for the *TMX3* gene and Orthologues**


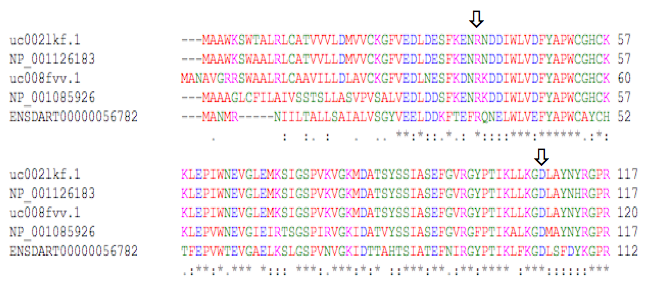


The amino acids p.39Arg and p.108Asp are indicated by the arrows. uc002lkf.1 = *H. Sapiens*, NP_001126183 = *P. Troglodytes*, uc008fvv.1 = *G. Gallus*, NP_001085926 = *X. Tropicalis*, ENSDART00000056782 = *D. Rerio*.
