# Supplementary material for: A Male with Unilateral Microphthalmia Reveals a Role for TMX3 in Eye Development
Source: PLoS One. 2010 May 11;5(5):e10565. doi: 10.1371/journal.pone.0010565 (PMC2868029; doi:10.1371/journal.pone.0010565)
Supplement: Table S2 — Non-coding sequence alterations in TMX3 in 162 patients with Anophthalmia/Microphthalmia. (0.05 MB DOC) [file pone.0010565.s003.doc]

**Table S2. Non-coding Sequence Alterations in *TMX3* in 162 patients with Anophthalmia/Microphthalmia**

| Nucleotide | Allele Frequency in 162 Patients with Anophthalmia or Microphthalmia | dbSNP^a^ | Allele Frequency from dbSNP |
| --- | --- | --- | --- |
| IVS1-25G>T | G = 0.997; T = 0.003 | - | - |
| IVS1+63C>T | C = 0.994; T = 0.006 | rs309249 | C = 0.991;  T = 0.009^b^ |
| IVS1+68T>A | T = 0.997; A = 0.003 | - | - |
| IVS2-63A>G | A = 0.997; G = 0.003 | - | - |
| IVS2+33T>C | T = 0.997; C = 0.003 | - | - |
| IVS2+122insT | Wildtype = 0.988;  InsT = 0.012 | - | - |
| IVS3+34C>A | C=0.975 A=0.025 | - | - |
| IVS3-34insT | Wildtype = 0.984;  IVS3-34insT = 0.016 | - | - |
| IVS4+11A>G | A = 0.997; G = 0.003 | - | - |
| IVS6+23T>C | T = 0.969; C = 0.031 | - | - |
| IVS6+35T>C | T = 0.849; C = 0.151 | - | - |
| IVS7+40A>C | A= 0.901; C=0.099 | rs433550 | A = 0.915;  C = 0.085^c^ |
| IVS8+67C>T | C=0.864; A=0.136 | rs412679 | - |
| IVS9+16delTT | Wildtype = 0.92  IVS9+16delTT = 0.08 | - | - |
| IVS10-22T>C | T = 0.997; C = 0.003 | - | - |
| IVS11-71A>T | A = 0.991; T = 0.009 | - | - |
| IVS13-145G>A | G=0.977; A=0.023 | - | - |
| IVS13-116G>A | G=0.96; A=0.04 | - | - |
| IVS13-104G>A | G=0.96; A=0.04 | - | - |
| IVS13+25G>C | G = 0.994; C = 0.006 | - | - |
| IVS13+26C>A | C = 0.994; A= 0.006 | - | - |
| IVS13+61A>G | A = 0.997; C = 0.003 | - | - |
| IVS14+91A>C | A = 0.98; C = 0.02 | - | - |
| IVS15+91C>T | C = 0.997; T = 0.003 | - | - |
| 3’UTR +43T>C | T = 0.98; C = 0.02 | rs309204 | T = 1.0^d^ |
| 3’UTR +52A>G | A = 0.997; G = 0.003 | - | - |

dbSNP^s^ = Database of Single Nucleotide Polymorphisms; b = Allele frequency in 116 CEU chromosomes; c = Allele frequency in 118 CEU chromosomes; d = Allele frequency in 112 CEU chromosomes.
